# The roughness of deciduous dentin surface and shear bond strength of glass ionomers in the treatment with four minimally invasive techniques

**DOI:** 10.1039/c9ra04159a

**Published:** 2019-10-09

**Authors:** María de los Angeles Moyaho-Bernal, Bitia Eunice Badillo-Estévez, Ester Luminosa Soberanes-de la Fuente, Maykel González-Torres, Bernardo Teutle-Coyotecatl, Gisela Nataly Rubín de Celís-Quintana, Rosendo Carrasco-Gutiérrez, Esther Vaillard-Jiménez, Gloria Lezama-Flores

**Affiliations:** Laboratorio de Biomateriales Odontológicos, Benemérita Universidad Autónoma de Puebla 72000 Mexico angeles.moyaho@correo.buap.mx lumisoberanes@hotmail.com moyaho3@gmail.com; Departamento de Odontología Pediátrica, Benemérita Universidad Autónoma de Puebla 72000 Mexico bitia.badillo25@gmail.com bertc_21@hotmail.com natalyrub@hotmail.com vaillarde@hotmail.com glorialezamaf@hotmail.com; Conacyt-Laboratorio de Biotecnología, Instituto Nacional de Rehabilitación “Luís Guillermo Ibarra” 14389 Mexico mikegcu@gmail.com; Tecnológico de Monterrey, Campus Ciudad de México 14380 Mexico maykel.gonzalez@conacyt.mx

## Abstract

The concept of minimally invasive technique in dentistry emphasizes conservative strategies in the management of caries, which results in less destruction of healthy tooth structure. The use of different techniques seems to interfere in the roughness of dentin and the mechanisms of adhesion with the restorative material. This study characterized the roughness of deciduous dentin surface treated with four minimally invasive techniques using profilometry, atomic force microscopy (AFM) and scanning electron microscopy (SEM); moreover, shear bond strength of Vitremer™ glass ionomer was determined. Samples were divided into four groups: G1_CB carbide bur, G2_PB polymer bur, G3_C Carisolv™, and G4_AA air abrasive. No differences were found between groups before and after treatment in the roughness. Samples treated with a carbide bur presented a smear layer; smart bur surface exhibited the remains of the material; G3_C Carisolv™ showed a rough surface, and air abrasive presented particle traces. Concerning the shear bond strength of Vitremer™ glass ionomer were not found differences after treatment (*p* > 0.05). It is concluded that roughness showed characteristic patterns derived from the technique used and the shear bond strength is not significantly affected after using any minimally invasive method.

## Abstract 2

The concept of minimally invasive technique in dentistry emphasizes conservative strategies in the management of caries, which results in less destruction of healthy tooth structure.

## Graphical abstract

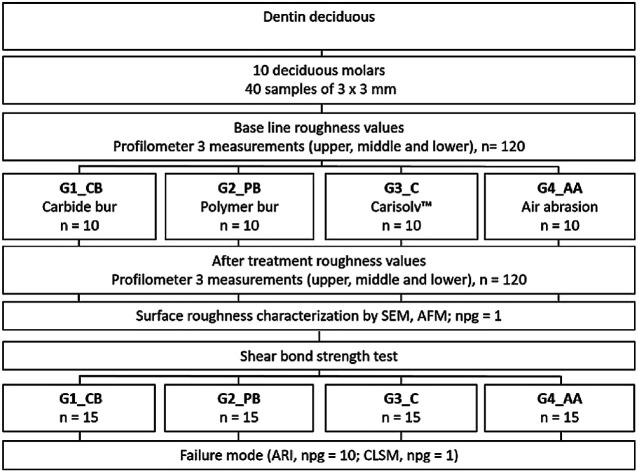

## Introduction

A thick dentin layer forms the bulk of mineralized dental tissues, mainly composed of type-I collagen fibrils and nanocrystalline apatite mineral.^1^ When dentin hydroxyapatite undergoes mineral loss, the demineralized substrate becomes rough and acquires high surface energy.^2^ These characteristics constitute significant factors that contribute to mechanical interlocking and the bond strength between restoration materials and dentin.^3^

Since 1957, mechanical rotary carbide burs have been used to eliminate carious lesions,^4^ but this technique suffers several problems derived from the sensitivity of vital dentin, the noise, and vibration generated by the bur, and the fact that it eliminates considerable amounts of healthy tissue unnecessarily.^5^ To overcome these disadvantages, an effective means of eliminating carious dentin is required that will promote adequate bond strength to restoration materials. As the concept of minimal intervention has gained ground in contemporary dentistry, more conservative approaches to caries elimination have been developed,^6^ aimed at removing carious tissue while preserving the dental structure as far as possible.^7^ In this context, several new minimally invasive techniques for carious lesion dentin removal have emerged, including:

• Polymer burs (smart burs) used for removing only infected dentin with the conservation of sound tooth structure. Its self-limiting capacity does not traumatize healthy dentinal tubules and reduces post-operative sensitivity.^8^

•Chemo-mechanical caries removal using hand instruments, in particular, Carisolv™, introduced in 1997,^9^ this technique is based on the degradation of carious tissue and in combination with sodium hypochlorite; it eliminates microorganisms and the affected dentin as the chlorine breaks down degraded collagen.^10^

•Air abrasion, originally developed by Black in 1945,^11^ removes tissue through the transfer of kinetic energy from incident particles traveling onto the softened dentin at high velocity, roughening the surface.^12^ Bioactive glass particles are deposited on the surface, favoring chemical interaction between dentin and the particles mineral content, which promotes remineralization, as well as desensitization effects. It is a biodegradable material whose degradation products do not have any cytotoxic effects on the organism.^13^

After treatment by minimally invasive techniques, deciduous dentin surfaces present specific roughness qualities. The resulting topography can have a profound effect on the ability of dental materials to bond to the treated surface and the technique used for carious lesion removal may be considered an important factor contributing to the bond strength between dentin and restorative materials.^14^ However, the surface topography/roughness generated by these techniques has not been extensively studied.

On the other hand, glass ionomers have occupied an important place in preventive and restorative dentistry. It is composed of an aluminum glass base, fluorine, calcium silicate or strontium combined with a water-soluble polyacrylic acid.^15,16^ However, this material has presented modifications not only in its composition and original chemical structure but also in its indications, clinical and aesthetic applications. Vitremer™ is a glass ionomer modified with a filling resin that it confers the peculiarity of being a material that resists the occlusal forces. One important question is whether there is any difference in the shear bond strength with the use of different methods of minimal invasion techniques in deciduous teeth. To the best of our knowledge, this research remains largely unstudied.

This work aimed to characterize the roughness of deciduous dentin and to determine the shear bond strength of Vitremer™ glass ionomer treated with minimally invasive techniques used for caries elimination.

## Materials and methods

### Tooth selection and sample preparation

The study protocol was approved by the Faculty of Dentistry Research Committee at the Meritorious Autonomous University of Puebla (Mexico). Forty, first and second deciduous molars extracted for orthopedic reasons, free of caries and without fillings, were obtained from patients, having gained informed consent from their parents. The teeth were cleaned with deionized water immediately and then stored in a 0.2 (wt/vol) thymol solution at 4 °C for up to 2 months. Sample preparation was performed by a single operator to prevent inter-operator variation. The teeth were sectioned at the cemento-enamel junction under deionized water irrigation to avoid dehydration of the organic component. A diamond disc (BesQual, New York, NY, U.S.A.) driven by a low-speed motor (Brasseler, Savannah, GA, U.S.A.) was used for sectioning. Then, the enamel was removed from the occlusal, mesial, distal, vestibular, and lingual/palatal surfaces to obtain dentin blocks measuring 3 × 3 mm. Afterward, the samples were observed under a light microscope (AXIO ZEIZZ Scope. A1, Germany) at 5× magnification to confirm the absence of enamel.

### Experimental groups

**Fig. 1:**
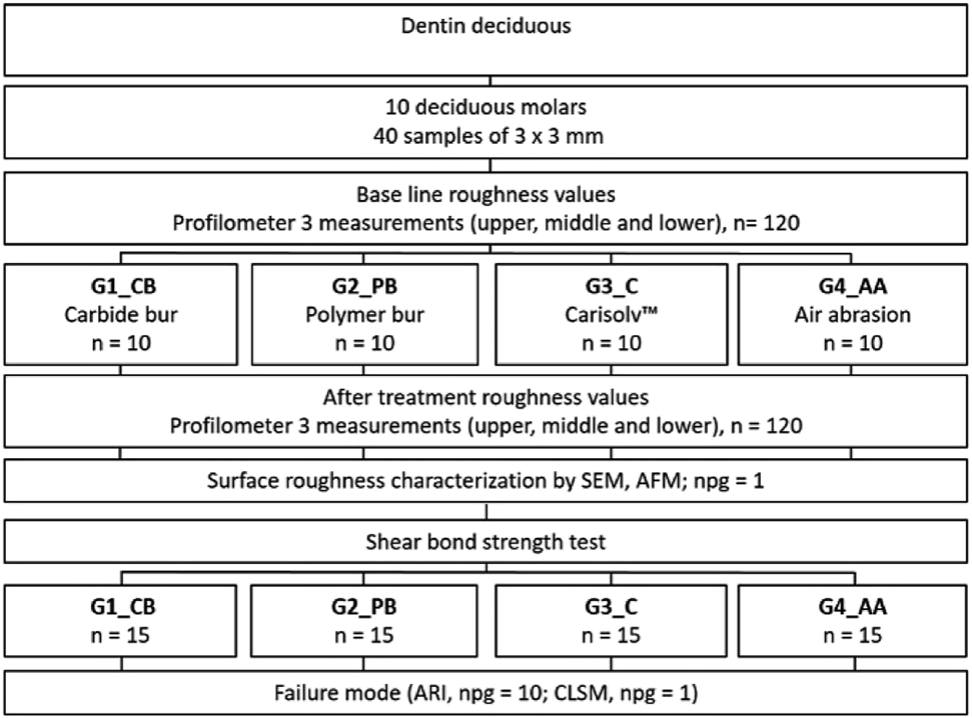
Schematic diagram showing the treatment groups.

Forty dentin samples were randomly divided into four groups (*n* = 10) to perform the different surface treatment techniques (Fig. 1). All deciduous dentin surfaces were treated by the same researcher to avoid inter-operator variation.

#### G1_CB

A rotary system (control group) was applied using a high-speed handpiece (W & H RC-90BC), with conventional tungsten carbide bur # 4 (MDT FG, TC0004, U.S.).

#### G2_PB

A smart bur technique with a low-speed contra-angle handpiece (W & H RC-90BC) and polymer bur # 4 (Smart Burs II SS White RA-4, U.S.).

The two techniques described above were applied over the occlusal surface at an angle of 0°, parallel to the tooth surface, and were repeated three times on the occlusal surface (mesial, medial and distal) accompanied by deionized water irrigation, both carbide and polymer burs were changed after treating each one sample, according to Wahle *et al.*, 1993.^17^

#### G3_C

Carisolv™ (MediTeam Dentalutveckling AB, Sweden), was applied following the manufacturer's instructions: one drop was placed on the surface with a syringe and tip supplied by the manufacturer, left on the surface for 30 seconds, and then washed with deionized water.

#### G4_AA

An air abrasive (Bioactive crystal particle) technique was carried out with the accessories provided by the manufacturer (CR SmarTip Sylc Denfotex, London). The particles were released from a distance of 1 mm onto the whole occlusal surface with a circular movement for 15 seconds (without irrigation).

After treatment, all samples were cleaned in an ultrasonic bath with deionized water for 10 minutes and carefully air-dried. Deciduous dentin roughness was characterized using a profilometer, observing four independent samples under AFM and SEM.

## Procedures

### Profilometry

The surface roughness of the dentin samples was measured before and after treatment with different minimally invasive techniques using a profilometer (Mitutoyo surf test SJ-301, Tokyo, Japan). The surface roughness of each sample was scanned at a length of 0.8 mm with a diamond stylus using a cutoff length of 0.08 (*λ*_c_). The scanned area was defined by the sample size (3 × 3 mm), measurements were carried out perpendicularly, and for each sample, three measurements were made by a single operator. Mean values were calculated for each sample and group. The roughness parameters assessed were the following: *R*_a_ (the arithmetic center-line average of the roughness profile) and *R*_z_ (peak-to-valley values of five equal lengths within the profile). All measurements were performed following ISO 4287-1997 guidelines^18^ (for surface texture: profile method).

### Atomic force microscopy

Four representative deciduous dentin samples (npg = 1) were examined using a scanning probe microscope (JEOL, JSPM-5200) to assess surface morphology in the tapping mode with a silicon nitride (Si_3_N_4_) probe with a tip height of 14 μm and <10 nm tip radius. The images were acquired at 256 × 256-pixel resolution and a scanning rate of 1 Hz for scanning square areas of 0.94 × 3.8 μm. The assessments were performed in the same room with temperature and relative humidity.

### Scanning electron microscopy

Four deciduous dentin samples (npg = 1) were fixed to aluminum stubs with double-sided adhesive carbon tape (SPI Supplies, United States). A scanning electron microscopy (JEOL, JSM-6610 LV, Japan) was used to obtain images of changes in roughness,^19,20^ with the following settings: low vacuum mode at 10 Pa chamber pressure, an electron acceleration voltage of 20 kV detecting backscattered electrons at a magnification of 1000×.

### Shear bond strength (SBS test)

Sixty samples were used following the same protocol described above. Then they were placed in acrylic blocks and divided randomly into four groups (*n* = 15), and the minimally invasive technique was applied concerning the assigned group. The Vitremer™ glass ionomer was placed in a press for Ultradent adhesion test (Ultradent products, USA) subsequently they were stored for 48 hours at 37 °C inside the incubator (Riosa E−41), finally were carried out to the universal test machine 4465 (Instron Corp., USA) to determine the SBS.

### Failure mode

Each sample was observed under a light microscope (AXIO ZEIZZ Scope. A1, Germany) to classify the type of adhesion failure at 35×. The Adhesive Remnant Index (ARI) was determined by the following criteria: 0 = no adhesive is present on the dentin surface, 1 = there is less than 50% remaining adhesive, 2 = more than 50% remaining adhesive, 3 = the entire dentine surface presents adhesive.

### The interface between dentin and restorative material

The samples were immersed for one minute in a sodium hypochlorite solution at 5%. After that, an ultrasonic bath of the samples was performed (Quantrex Q140 L & R Ultrasonic, NJ, USA) for 10 minutes with deionized water, were dried at room temperature. Then, it was added to a rhodamine B solution (5 × 10^−4^ g ml^−1^), covered with aluminum foil and left for 24 hours at 4 °C; subsequently, it was washed with a PBS solution, shaken with the vortex mixer for 5 minutes and dried at room temperature until use. Immediately, Confocal Laser Scanning Microscope (CLSM) (Carl Zeiss LSM5 PASCAL Azioskop 2M; excitation wavelength of 640 nm, with magnifications of 4× and 10×) was used to observe the interface between dental and restoration material, viewed longitudinally.

### Statistical analysis

The Shapiro Wilk normality test was applied to all variables, and the paired *T*-test was used to compare the roughness before and after treatments. One-way analysis of variance (ANOVA) was performed to compare treatment groups as well as for shear bond strength. The ARI was evaluated by the Kruskal–Wallis test. All data were analyzed using the SPSS version 21 (SPSS, Inc., Chicago, IL, U.S.A.) statistical software package, with statistical significance established at *p* <0.05.

## Results

### Surface roughness

For each parameter measured, mean values were calculated before and after treatment. Generally, the roughness was observed to be homogeneous (Shapiro–Wilk test; *p* <0.05) before treatment with an increase in roughness values after treatment. A paired *T*-test was applied to compare data before and after treatment, finding statistically significant differences (*p* < 0.05). Levene's test and one-way ANOVA were used to compare groups, but no statistically significant differences were found. Table 1 shows the numerical analysis of the surface roughness parameters of the 40 samples evaluated.

The roughness of deciduous dentin before and after minimally invasive treatments

**Table:** 

Groups (*n* = 10)	*R* (μm)	
	*R*_a_ (μm) BT	*R*_a_ (μm) AT
G1_CB	0.51 ± 0.24 A a	2.12 ± 0.64 A b
G2_PB	0.59 ± 0.28 A a	2.10 ± 1.04 A b
G3_C	0.56 ± 0.18 A a	1.95 ± 0.65 A b
G4_AA	0.68 ± 0.26 A a	2.21 ± 0.95 A b

**Table:** 

	*R*_z_ (μm) BT	*R*_z_ (μm) AT
G1_CB	4.59 ± 2.14 A a	11.35 ± 3.82 A b
G2_PB	4.77 ± 2.34 A a	12.89 ± 6.16 A b
G3_C	4.70 ± 1.66 A a	11.76 ± 3.61 A b
G4_AA	6.00 ± 2.05 A a	12.21 ± 4.02 A b

Capital letters in a column are the comparison between the roughness values of different groups. Same capital letters follow means that do not differ statically [one-way analysis of variance (ANOVA), *P* < 0.05]. Lower-case letters in a row are for the comparison of roughness before and after of treatment. Lower-case letters follow means that do not differ statistically [paired-samples *t*-test, *P* < 0.05].BT, before treatment; AT, after treatment.

### Atomic force microscopy

**Fig. 2:**
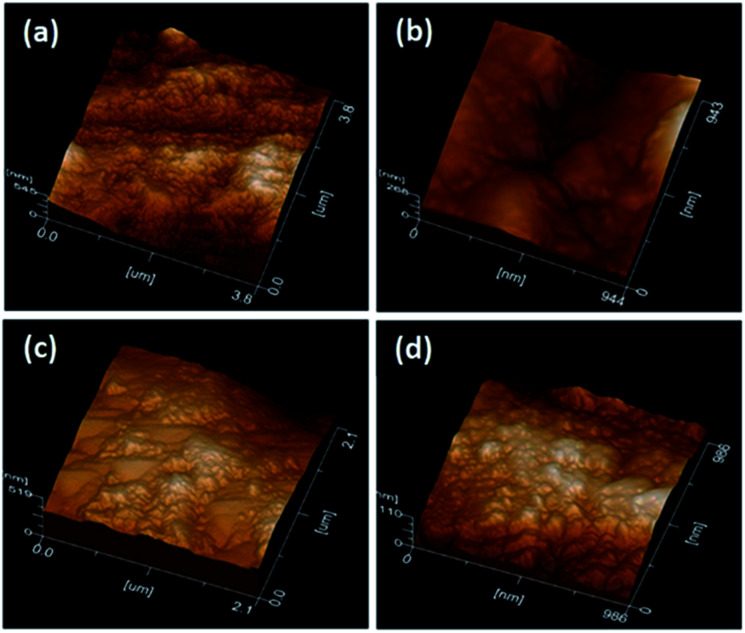
AFM image after minimally invasive techniques.

The deciduous dentine surfaces presented characteristic patterns according to the technique used. G1_CB presented homogeneous roughness (Fig. 2a), G2_PB showed an indefinite structure, greater roughness, without a well-defined pattern (Fig. 2b), G3_C exhibited some irregular, and flat zones (Fig. 2c) and G4_AA displayed a surface with a uniform pattern of roughness (Fig. 2d).

### Scanning electron microscopy

**Fig. 3:**
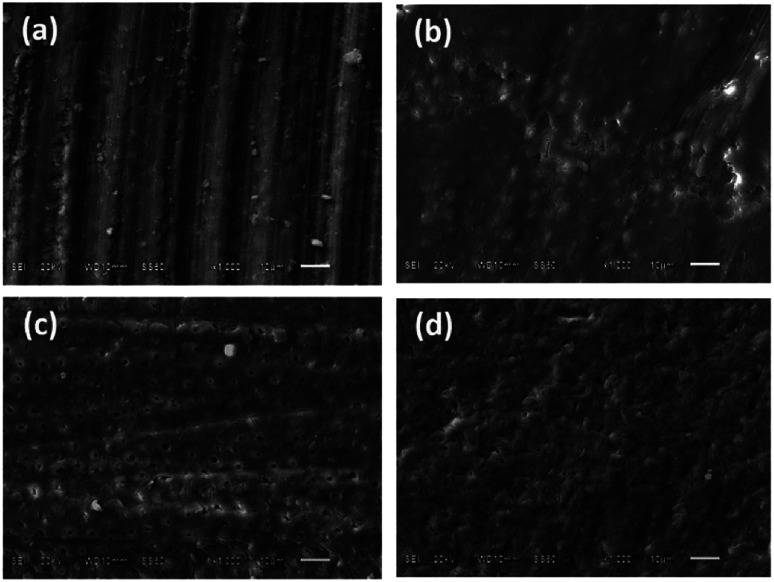
Representative Scanning Electron Microscopy micrographs of dentin surface (barcode: 10 μm).

The morphology of deciduous dentin varied distinctly according to the treatment technique used. G1_CB showed closed dentinal tubules due to the presence of a smear layer covering the dentin surface (Fig. 3a); G2_PB presented an indefinite structure with obstructed dentinal tubules, a few cracks in some areas, with the presence of polymer bur debris (Fig. 3b); G3_C displayed an irregular surface with the presence of an amorphic layer, like a smear layer, and in a few areas, there were exposed dentinal tubules with different diameter sizes (Fig. 3c) and G4_AA exhibited an extremely irregular surface with some exposed dentinal tubules and the presence of remains of bioactive crystal particles (Fig. 3d).

### Shear bond strength (SBS test)

The samples were immediately tested for SBS in the universal testing machine, all groups showed normal distribution (*p* > 0.05), except in G2_PB (*p* < 0.05). One-way ANOVA was applied, and the results showed that treatment in G3_C was the highest; while G2_PB presented the lowest value. However, there was no significant difference between groups (Table 2).

**Table:** Shear bond strength values after the minimally invasive techniques employed^a^

Group (*n* = 15)	MPa	ANOVA
G1_CB	11.49 ± 4.08	NS
G2_PB	10.68 ± 5.32	
G3_C	14.92 ± 4.37	
G4_AA	12.20 ± 6.32	

a Mean values: NS represented no significant statistics.

### Adhesive remnant index

The samples were observed under a light microscope and presented ARI values from 0–2. The ARI = 3 score was not found in any of the samples. The G1_CB and G2_PB there was no remaining material on the surface, G3_C displayed a predominance of remnant on the surface of more than 50%. The G4_AA presented less than 50% of the remnant (Table 3).

**Table:** ARI scores for each group and their respective frequency and percentage^a^

Group (*n* = 10)	ARI score		
	0	1	2
G1_CB	5 (50%)	4 (40%)	1 (10%)
G2_PB	6 (60%)	4 (40%)	0 (0%)
G3_C	2 (20%)	4 (40%)	4 (40%)
G4_AA	4 (40%)	5 (50%)	1 (10%)

a Score 0: no adhesive is present on the dentin surface, 1 = there is less than 50% remaining adhesive, 2 = more than 50% remaining adhesive.

El G3_C presented the highest, while G2_PB the lowest average; however, no statistically significant differences were found between the four groups (Table 4).

**Table:** Comparison of the ARI index between groups^a^

ARI score		
Group (*n* = 10)	Mean	Kruskal Wallis
G1_CB	0.6 ± 0.70	NS
G2_PB	0.4 ± 0.52	
G3_C	1.2 ± 0.79	
G4_AA	0.7 ± 0.67	

a Mean and standard deviation values. NS represented no significant statistics.

### Confocal laser scanning microscopy

**Fig. 4:**
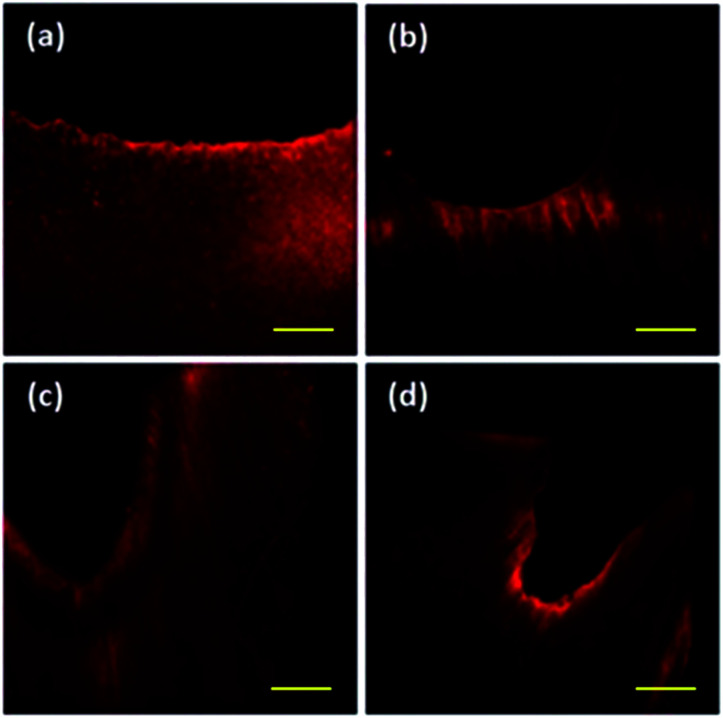
CLSM views of deciduous dentine surfaces treated (barcode: 100 μm).

The highest concentration of staining with rhodamine B is found in the restorative material. The G1_CB presented more uniform staining (Fig. 4a) as compared with G2_PB, where a well-delimited area with a very thin and shallow thickness was observed (Fig. 4b). The G3_C depicted a greater penetration thickness of rhodamine B (Fig. 4c). The G4_AA revealed heterogeneous zones with different thicknesses (Fig. 4d).

## Discussion

Although minimally invasive techniques are commonly used in pediatric dentistry, their effects on deciduous structures have not been investigated. In this context, the morphology of dentin is one of the main factors determining the success of bonding to dentin. The present study focused on characterising the roughness of dentin surfaces treated with the difference of minimally invasive techniques in current use for the elimination of carious lesions. Several *in vitro* studies have assessed the roughness of dentin surfaces after using the carbide bur as conventional technique^14,21^ polymer bur,^22^ Carisolv™^14,23,24^ air abrasive,^25^ sono-abrasion^14^ and also Er: YAG laser irradiation.^14,21^ However, clear comparisons between minimally invasive techniques remain scarce and have been limited to permanent teeth.

It was decided to evaluate the conventional carbide bur as a mechanical method of removing carious lesions (control group), since it has been used since 1957.^4^ According to the manufacturer, the polymer burs investigated are made from a special polymer material, which offers the advantage of cutting fewer dentin tubules, and it causes less pain than conventional burs.^8^ The chemical–mechanical removal method tested in the present study, Carisolv™, is not cytotoxic and shows acceptable biocompatibility with oral cells.^26^ Moreover, materials such as the crystal particles based on bioactive substances assessed, have the potential to promote dentin remineralization due to their notable bioactive capability to form hydroxycarbonate apatite (HCA).^27,28^

### Deciduous dentin surface roughness characterization

The study used three methods for surface characterization: a quantitative method (profilometry) and two qualitative ones (AFM and SEM), in order to obtain relevant information in addition to profilometry. Roughness was measured before treating the surfaces and observed homogeneous surfaces. After applying the different treatment techniques, surfaces exhibited significant increases in roughness with values that were influenced by the instrument (carbide bur and polymer bur) or material used (Carisolv™ and bioactive crystal particles). However, no significant differences were observed in roughness data between the techniques, regardless of the roughness parameter used for evaluation. Wahle and Stanley,^17^ concluded that a carbide bur creates variable surface roughness depending on the brand of bur used. Gheorghiu *et al.*,^22^ found that the surface roughness obtained was more pronounced than that obtained with a polymer bur, but this finding disagrees with the present study. Wennerberg *et al.*,^23^ found that Carisolv™ produced a smoother surface than conventional caries removal using carbide burs. However, it is difficult to make direct comparisons between our results and previous studies due to the variability in sample processing and the techniques used. In any case, the literature includes very few similar investigations, and there is not much information available for comparison.

### Atomic force microscopy and scanning electron microscopy

AFM and SEM were found to be useful techniques for visualizing the surface morphology of the roughened surfaces produced by the different treatment techniques. Conventional bur excavation results in rapid and excessive removal of uninfected dentin.^29,30^ We observed that resulting surface typically exhibited a homogeneous and flat appearance covered with a debris-like smear layer; roughness was uniform with no opening of the dentinal tubules, observations that concur with Yazici *et al.*^14^

### Minimally invasive techniques

According to the product specifications issued by the manufacturer, the polymer bur (Smart Bur) is composed of polyamide, which is harder (Knoop hardness 50) than decayed dentin (Knoop 10–40), but not as hard as sound dentin (Knoop 70–90).^8^ This property makes it possible to remove dentin tissue affected by caries safely without eliminating healthy dentin, however, were observed surfaces treated with the polymer bur exhibited a pattern of relatively smooth appearance and only small irregularities, a few cracks,^22^ as well as remains of the polymer bur used.^14^

It is desirable to conserve dental structures as far as possible; the new cavity preparation techniques aim to minimize the scale of intervention, the amount of tissue removal, as well as patient discomfort. In this sense, they are particularly suited to pediatric patients. Such techniques are known as minimal intervention.^29^

Among these recently developed techniques, the chemo-mechanical method Carisolv™ is a promising method for treating deciduous teeth due to its conservative and pain-reducing characteristics, which compare favorably with conventional drilling for caries removal. This study found that treatment with Carisolv™ produced an irregular surface with the presence of an amorphic layer, like a smear layer, and in a few areas, there were exposed dentinal tubules with different diameter sizes^14,31,32^ This may be due to its sodium hypochlorite content.^9,33^ The mechanism of action for dissolving organic tissue is considered to require a pH of around 12 and it is postulated that positively and negatively charged groups of amino acids become chlorinated and further disrupt the collagen cross-linkage in the matrix of carious dentin, dissolving the caries-infected dentin as chlorine breaks down the triple helix of polypeptide collagen chains between the fibers.^10^ This process results in a rough surface, with a ‘melted’ appearance^31,32^ and a substrate with high surface energy.^2^ Some studies have noted that deproteinized dentin presents greater hardness,^26^ a higher elastic modulus,^34^ greater wettability^35^ and permeability than demineralized healthy dentin. However, there is little information of this type about deciduous dentin. Some authors have argued that demineralizing dentin can interfere with adhesion,^36^ but other authors have noted that its substrate characteristics can produce an increase in the bond strength of adhesive systems applied over the deproteinized substrate.^37,38^ It would appear that adhesion to dentin depends on both the adhesive system used and the characteristics of the dentin substrate.^39^

The air abrasive method has a less aggressive cutting action; it is subject to several variables such as powder flow rate, particle size, and exit pressure, all of which have a marked effect on the efficiency of their when compared with other minimal intervention techniques.^13^ Bioactive crystal resulted in exposed dentinal tubules, and the presence of particle remains,^14^ according to some authors, the technique has a beneficial effect on dentin,^13^ forming hydroxycarbonate apatite (HCA).^27,28^

### Shear bond strength (SBS test)

There is a wide variety of options available for carious lesion removal, and each creates a different type or degree of roughened dentin surface. In addition to the choice of technique and the surface it produces (smart bur,^22^ Carisolv™,^40^ bioactive glass particles),^13^ the use of new restoration materials is also an important factor determining bond strength. Each must be selected according to the clinical needs of the individual pediatric case. For this reason, there is a need for greater understanding of how the surface produced interacts with the restoration technique used; the combination of minimally invasive techniques and restoration materials will act in synergy to optimize bond strength.^36–38^

It is of note that the deviation of the shear bond strength values in Table 2 are high. This variability can result from many different sources, including specimen preparation.^41,42^ However, the study was controlled by the use of a standardized protocol, and all deciduous dentin surfaces were treated by the same researcher to avoid inter-operator variation. Other possible factors could be the techniques and products employed;^41^ nevertheless, they were applied following the manufacturer's instructions. Another option could be the differences in the deciduous dentin; unfortunately, the substrate-related^42^ variables are more difficult to control due to the nature of teeth, storage conditions of the bonded samples; however, the storage effects on dentin permeability and shear bond strengths were previously analysed.^43^ The authors concluded that bond strengths were unaffected by duration of storage or by solution type, except for saline. We decided to store the teeth in a 0.2 (wt/vol) thymol solution at 4 °C for up to 2 months. Conclusively, the high standard deviation is primarily associated with alignment problems during the debonding test due to failures occurring at low-stress levels or before the specimens could be tested in the testing machine.

One of the main requirements for adhesion is the adaptation of the restorative material to the tooth structure, and it was decided to use the confocal microscope to describe the interface between the glass ionomer and the dental surface as it is one of the least destructive methods for organic structures. Rhodamine B is used because of its compatibility as Watson suggested.^44^ The adhesion of the Vitremer™ glass ionomer was observed with a suitable adaptation as previously reported.^45^

### Dental material

Regarding the material, it was decided to use the Vitremer™ since it is a new material widely used in pediatric dentistry to restore cavities, in addition to its conditioning system due to the micromechanical and chemical interaction with the dental substrate; therefore, it respects the dental structure that remains after the use of any procedure.

According to the manufacturer's instructions, the glass ionomer Vitremer™ can restore cavities class I, II, III, and V^46^ but most of the studies reported do not consider the structure of the deciduous teeth for evaluation in terms of sealing and adhesion strength.

Resistance values of resin-modified glass ionomer restorations reported in the literature are difficult to compare due to the great variability of composition, manufacturing process, particle size of the powder, concentration and molecular weight of the liquid, as well as the conditions at the time of the test that may interfere in the results,^47^ however, the procedures in this investigation were adhered to according to the provisions of ISO/TS 11405-2015.

In spite of the optimal conditions of the procedures, no significant differences were found in shear bond strength between the different techniques studied, as reported by Hamama *et al.*^48,49^ However, the Carisolv® method showed the highest adhesion values concerning the analysis of the failure modes (ARI). It also presented the highest percentage of adhesive remaining in the dentin surface. This result can be attributed to the properties of the material that only degrades the damaged tissue.^50^

## Conclusions

According to the results, it may be concluded that: (a) there is a significant difference in surface roughness resulting from treatment by the four minimally invasive methods compared; however, no significant differences in roughness were found between the techniques. Therefore, anyone of them can be recommended, (b) under the AFM and SEM examination, the surface topography of the treated dentin showed characteristic patterns derived from the particular technique used (c) the shear bond strength of the glass ionomer is not significantly affected when using any minimally invasive method.

## Ethical statement

All experiments were performed in accordance with the Guidelines of Council far lnternational Organizations of Medical Sciences in collaboration with the World Health Organization: CIOMS/WHO (2002), and to parameters of medical research manifested by the World Medical Association (WMA), WMA (2009), as well as adhering to the General Law on Research Health. Experiments were approved by the ethics committee at BUAP. Informed consents were obtained from human participants of this study.

## Conflicts of interest

There are no conflicts to declare.

## Supplementary Material
